# Comprehensive SNP Scan of DNA Repair and DNA Damage Response Genes Reveal Multiple Susceptibility Loci Conferring Risk to Tobacco Associated Leukoplakia and Oral Cancer

**DOI:** 10.1371/journal.pone.0056952

**Published:** 2013-02-20

**Authors:** Pinaki Mondal, Sayantan Datta, Guru Prasad Maiti, Aradhita Baral, Ganga Nath Jha, Chinmay Kumar Panda, Shantanu Chowdhury, Saurabh Ghosh, Bidyut Roy, Susanta Roychoudhury

**Affiliations:** 1 Cancer Biology and Inflammatory Disorder Division, CSIR-Indian Institute of Chemical Biology, Kolkata, West Bengal, India; 2 Human Genetics Unit, Indian Statistical Institute, Kolkata, West Bengal, India; 3 Oncogene Regulation and Viral associated Human cancer, Chittaranjan National Cancer Institute, Kolkata, West Bengal, India; 4 Proteomics and Structural Biology Unit, CSIR-Institute of Genomics and Integrative Biology, New Delhi, India; 5 Department of Anthropology, Vinoba Bhave University, Hazaribag, Bihar, India; University of Pittsburgh, United States of America

## Abstract

Polymorphic variants of DNA repair and damage response genes play major role in carcinogenesis. These variants are suspected as predisposition factors to Oral Squamous Cell Carcinoma (OSCC). For identification of susceptible variants affecting OSCC development in Indian population, the “maximally informative” method of SNP selection from HapMap data to non-HapMap populations was applied. Three hundred twenty-five SNPs from 11 key genes involved in double strand break repair, mismatch repair and DNA damage response pathways were genotyped on a total of 373 OSCC, 253 leukoplakia and 535 unrelated control individuals. The significantly associated SNPs were validated in an additional cohort of 144 OSCC patients and 160 controls. The rs12515548 of *MSH3* showed significant association with OSCC both in the discovery and validation phases (discovery P-value: 1.43E-05, replication P-value: 4.84E-03). Two SNPs (rs12360870 of *MRE11A*, P-value: 2.37E-07 and rs7003908 of *PRKDC*, P-value: 7.99E-05) were found to be significantly associated only with leukoplakia. Stratification of subjects based on amount of tobacco consumption identified SNPs that were associated with either high or low tobacco exposed group. The study reveals a synergism between associated SNPs and lifestyle factors in predisposition to OSCC and leukoplakia.

## Introduction

Oral squamous cell carcinoma (OSCC) is the tenth most common cancer worldwide. In India, OSCC ranks first among men and fourth among women [1], [2]. The oral cavity regions that are affected by this cancer are tongue, buccal mucosa, lip and gingiva. Known risk factors for oral cancer are tobacco chewing and smoking, alcohol consumption, HPV infection and gender [3]. The incidence (9.8 in men, 5.2 in female per 10,0000 persons per year) and mortality rate (22.1 in men, 9.4 in female per 100,000 persons per year) of OSCC escalated in Indian populations due to an increasing rate (35%) of tobacco consumption [4], [5]. Among various provinces of India, West Bengal population has high age standardized tobacco related cancer mortality rate (33.4) and cumulative risk 5.0% (99% confidence interval [CI] 4.1–5.8) [2]. The most common clinically presented premalignant lesion of buccal mucosa is oral leukoplakia with a prevalence of 0.1–0.5% and rate of transformation to cancer is 1–2% per year [6]. The treatment and assessment of the risk and progression of leukoplakia remains a problem, as it recurs despite of its removal via surgery, and chemotherapy does not decrease cancer incidence [7]. Thus, genetic marker based risk assessment is necessary for early detection.

Numerous genetic association studies have identified SNPs in several important genes like *p53, p73* and *MDM2* as risk factors to OSCC and leukoplakia development [8]–[10]. A Genome Wide Association Study (GWAS) on Upper Aerodigestive Tract Cancers (UADT), that included the oral cavity regions, identified susceptible variants mainly in *aldehyde dehydrogenase* (*ADH*) gene cluster [11]. Cellular DNA repair processes stabilize the genome by reducing carcinogen induced mutations [12]. However, studies on repair gene variants and OSCC susceptibility focused mainly on *XRCC* group of genes and on few other DNA repair associated genes like *ATM*, *NBN* and *MRE11A* in different populations worldwide [13], [14]. In Indian populations, association of polymorphisms in *XRCC1, XRCC3, NAT2, XPD, ERCC2* and *OGG1* with OSCC have been reported [15]–[19].

Double Strand Breaks (DSB), is considered to be the most lethal among the different kind of damages [20] and Non Homologous End Joining repair (NHEJ) is the major pathway for the DSB repair process [21]. The other DNA repair pathway that has been reported to be compromised in OSCC is the MisMatch Repair (MMR) pathway. The members of MMR play important roles in reducing the mutation rate and genomic instabilities [12]. *MLH1* and *MSH2* of MMR are inactivated by promoter hyper-methylation in OSCC [22]. These DNA repair genes are also major targets for many anti-cancer drug development studies [23], [24]. Polymorphisms in these genes modulate the individual response to carcinogenic agents [25] and to drugs [26], [27].

We performed a case-control association study to identify risk SNPs at major DSB repair (*RAD50, MRE11A, NBS1, PRKDC, XRCC5, XRCC6* and *LIG4*), MMR (*MSH6* and *MSH3*) and key DNA damage response (*ATM* and *ATR*) genes in oral cancer and leukoplakia patients from the state of West Bengal of eastern India. In the discovery phase we genotyped 321 SNPs in 626 cases (373 individuals with OSCC and 253 individuals with leukoplakia) and 535 age-matched controls with similar tobacco smoking and/or chewing habits and no oral ailments. Subsequently, we validated significantly associated SNPs in a separate replication cohort of 114 OSCC patients and 160 controls from the same geographic locations. Finally, we performed a Multi Dimensionality Reduction (MDR) analysis to observe SNP-SNP and SNP-environment interaction.

## Methods

### Ethics Statement

Procedures for collection of blood samples and written informed consent form were reviewed and approved by the Institutional Ethical Committee, CSIR-Indian Institute of Chemical Biology, Kolkata, India.

Written informed consent was obtained from all case and control subjects after explaining the collection procedures and purpose of the study in local languages.

### Subjects

In the discovery phase, 373 OSCC and 253 leukoplakia patients were recruited between 2006 and 2009 from R. Ahemed Dental College and Hospital, Kolkata India after pathologist from the hospital confirmed these two types of lesion by histo-pathological examinations. These patients are caste populations of low and middle income group (annual family income <$100 and <$300, respectively) from various districts of the state of West Bengal in the eastern region of India. We, therefore, recruited 535 ethnically matched but unrelated control individuals either from the same hospital who have come to the hospital for dental and oral check up and have no oral ailments and also directly from the population by visiting various locations of the state of West Bengal. The potential consequence of using hospital based control is biased sampling which we have tested by principal component analysis and adjusted the bias, if any. Control individuals recruited from population were examined by physicians to ensure that individuals without any oral ailments are enrolled. Both patients and controls were regular tobacco users, either in the form of smoking and/or chewing, at the time of collection. We divided both patients and controls based on tobacco exposure level: (a) High Dose (HD) and (b) Low Dose (LD) tobacco exposed groups. We computed tobacco smoking and chewing index, PY (Pack Year) and CY (Chewing Year), respectively by using the following formula as used in earlier studies: (No. of cigarettes per day/20× No. of years)+(No. of bidis per day/40× No. of years) for PY and (No. of times per day × No. of years) for CY [28]. Next, we used median values of PY and CY to divide the subjects in HD and LD groups. In the replication phase, another 114 OSCC patients from Chittaranjan National Cancer Research Institute, Kolkata, India and 160 controls were recruited with the same inclusion and exclusion criteria. Fresh 5–10 ml blood samples were collected with informed consent from patients and controls. Information on age, sex, oral hygiene, tobacco habits and alcohol consumptions were recorded by interviewing both patients and controls.

### Genes and SNP Selection

We selected seven key genes for selection of SNPs from DSB repair pathway (*LIG4, MRE11A, PRKDC, NBN, RAD50, XRCC5* and *XRCC6*), two major genes from MMR pathway (*MSH6* and *MSH3*) and two genes from DNA damage response pathway (*ATM* and *ATR*). We chose all genes of NHEJ core repair machinery as it is the major repair process of DSB pathway and also remain active throughout the cell cycle compared to homologous recombination repair process [21], [29]. The core component includes XRCC5 and XRCC6 that form a dimer and together with PRKDC recognize the double strand breaks. Subsequently, the MRN complex composed of MRE11A, RAD50 and NBN clean up the ends and finally LIG4 seals the gap [29]. In mismatch repair pathway, we focused our study on mismatch recognition process. Two different complexes composed of MSH2-MSH3 and MSH2-MSH6 recognizes mismatches and Insertion/Deletion Loops (IDLs), respectively. Although many genetic association studies have been performed in *MSH2* in oral and colorectal cancer, the genetic association of *MSH3* and *MSH6* in different cancers is only beginning to be understood [30]–[34]. The *ATM* and *ATR* genes selected from the DNA damage response pathway as these genes are major signal transducers that initiate DNA damage related signalling for repair [35], [36]. A “maximally informative” method of SNP selection from HapMap data to non-HapMap populations was applied to select SNPs [37]. For easy understanding, we have provided the details and step by step process of this selection algorithm with the permission of the authors in online supplementary methods (Methods S1). The list of SNPs was submitted to Illumina to estimate the GoldenGate assay success rate and finally 321 SNPs were selected for discovery phase analysis. In the replication phase we genotyped only those SNPs that showed significant association with the OSCC development (P-value <0.05). Replication of the SNPs that were found to be associated with the leukoplakia could not be done due to unavailability of a new cohort of these patients in sufficient numbers.

### Genotyping, Quality Control and Statistical Methods

Genomic DNA was isolated from peripheral blood leukocytes using the QIAGEN blood DNA isolation kits as per manufacture protocol. The concentration of DNA samples were estimated by picogreen assay and diluted to a concentration of 50 ng/µL. The Illumina GoldenGate assay (Illumina, San Diego, USA) was used for genotyping in the discovery phase and in the replication phase genotyping was performed by TaqMan assay in real time PCR machine 7500 Fast and StepOne Plus (Applied Biosystems, Foster City, USA). Both kind of genotyping were performed as per manufacture’s protocol and we included 10% samples as replicate in each platform to measure genotyping replication error. For GoldenGate assay, we discarded data with a GenCall score <0.25 as the potential outliers and checked controls and contamination dashboards for each plate. For TaqMan, we used automated clusters and checked FAM and VIC dye intensities, and cycle threshold values in each plate. The software used for genotype call were Illumina’s BeadStudio (version 2.3.43), StepOne (version 2.2) and 7500 SDS (version 2.0.5).

**Figure 1:**
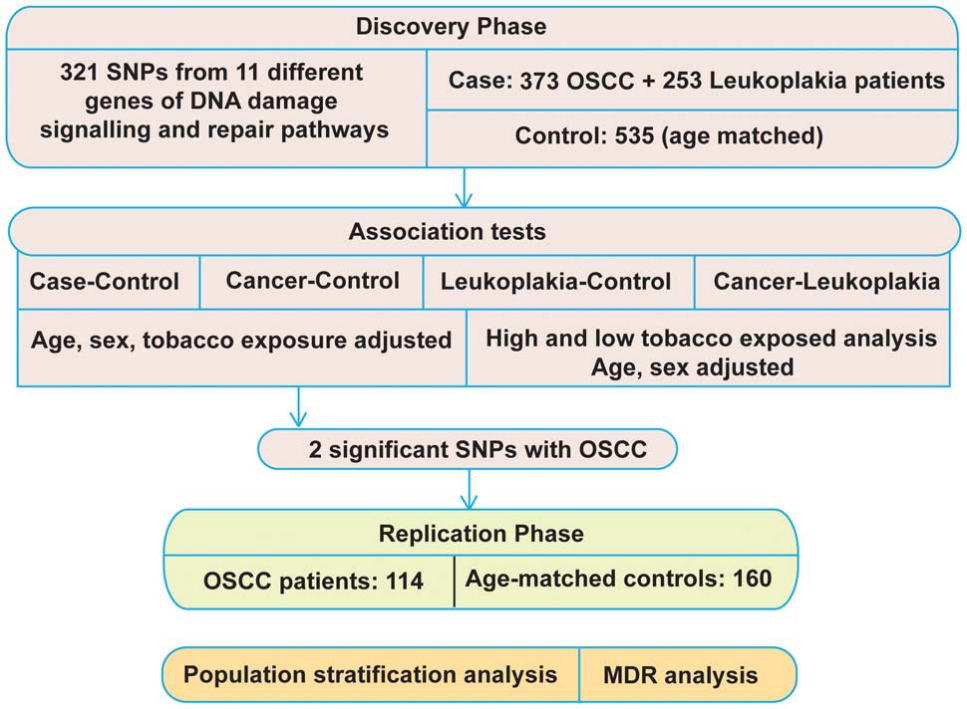
Overall strategy of the association study.

To ensure high quality data in the final association analysis, we discarded data on (a) SNPs that did not have valid genotype calls on >90% of sampled individuals, and (b) individuals for whom genotype calls on >8% of the SNPs were missing. Further, data on SNPs for which the Minor Allele Frequency (MAF) was <0.05 and had a P value <0.001 for departure from Hardy-Weinberg equilibrium were also discarded. The study design is presented in Fig. 1. The allelic and genotypic association tests were performed in four different ways: (a) Case versus Controls (CC), where case included both OSCC and leukoplakia samples; (b) Cancer versus Controls (CAC), where only OSCC samples were considered as cases; (c) Leukoplakia versus Control (LC) and (d) Cancer versus Leukoplakia (CAL), where leukoplakia samples were considered as controls. In each set, P-values, odds ratios (OR) and 95% CI were determined by logistic regression using age, sex and tobacco habits as covariates. Finally, all the unadjusted P-values were corrected for multiple testing by Benjamini-Hochberg step up False Discovery Rate control (FDR-BH) [38]. Additionally, to eliminate any population stratification effect on the association tests, we performed Identity-by-State (IBS) clustering of the genotyped data and generated first four principal components. All four components of PCA (Principal Component Analysis) were then used as covariates along with other covariates as mentioned earlier for allelic and genotypic association testing [39].

As tobacco habit is strongly associated with cancer development, we also performed association analysis using tobacco smoking and chewing as covariates in logistic regression. Subjects were divided into high dose (HD) and low dose (LD) as described above. Association P-value of the HD and LD groups were also adjusted for age and sex by logistic regression and corrected by FDR-BH.

Association tests, logistic regression, multiple testing corrections and PCA were performed using PLINK [40]. The PCA data was visualized by R [41], Mann-Whitney and chi-square tests in Table 1 and Table 2 were performed online at http://faculty.vassar.edu/lowry/utest.html and http://www.graphpad.com/quickcalcs/contingency1.cfm, respectively. The power of the study is calculated from http://www.stat.ubc.ca/~rollin/stats/ssize/caco.html.

**Table 1:** Basic characteristics of case and control data in discovery phase.

Parameters		Con (n = 535)	Case (Can+ Leu) (n = 625)	Can (n = 373)	Leu (n = 253)	P-value			
						Case- Con	Can - Con	Leu- Con	Can - Leu
**Age**	**Range**	22–85	20–88	25–88	20–75	<0.001^a^	<0.001^a^	0.458^a^	<0.001^a^
	**Median**	48	50	55	46				
**Sex**	**Male**	379	443	230	213	0.353^b^	0.103^b^	<0.001^b^	<0.001^b^
	**Female**	156	160	121	39				
	**M:F Ratio**	2.42	2.76	1.9	5.46				
**Pack Year**	**Range**	0.13–98.33	1–90	1–75	1–90	0.175^a^	0.001^a^	<0.001^a^	<0.001^a^
	**Median**	15	15	15	15				
**Chewing Year**	**Range**	0.51–960	3–1000	4–1000	3–720	0.153^a^	<0.001^a^	<0.001^a^	<0.001^a^
	**Median**	115	111.5	120	80				

Abbreviation: Con: Control, Can: Oral cancer, Leu: Leukoplakia.

a P-values from Mann-Whitney test,

b p-values from chi-square test.

**Table 2:** Basic characteristics of cancer and control in replication phase.

Parameters		Control(n = 160)	Cancer(n = 114)	P-value
**Age**	**Range**	20–80	27–81	0.221^a^
	**Median**	51	53	
**Sex**	**Male**	116	76	0.3491^b^
	**Female**	44	38	
	**M:F Ratio**	2.636	2	
**Pack Year**	**Range**	1–74.12	0.63–71	0.917^a^
	**Median**	15	16.5	
**Chewing Year**	**Range**	1–980	3.45–893.5	0.733^a^
	**Median**	120	100	

Abbreviation:

a p-values from Mann-Whitney test,

b P-values from chi-square test.

### MDR Analysis of SNP-SNP and SNP-environment Interaction

To analyze possible interaction among the associated SNPs and all the covariates, we used the non-parametric MDR approach, as described previously [42]. MDR, a constructive induction process [43], defines a single variable that incorporates information from multi locus genotypes and other disease controlling factors and store as either high or low disease risk group. We included significant SNPs and all covariates (Age, Sex, PY and CY) to construct interaction models separately in CC, CAC, LC and CAL groups. Statistical significance was determined using permutation testing in MDRpt (version 1.0_beta_2). We used 10 fold cross-validation and 1000 fold permutation testing and considered those interaction models as significant which showed a P-Value less than 0.05. Among the significant models, we identified important ones which have a cross validation consistency (CVC) ≥9, as the data was cross validated 10 times by MDR. The best model was then defined with the largest testing balance accuracy (TBA) among the important models. The MDR and MDRpt are open-source software and freely available from http://www.epistasis.org.

We also build hierarchical interaction entropy graphs to quickly access and interpret MDR models based on the theory of information gain as described previously [44] using Orange software package [45].

## Results

### Sample Ascertainment

We have presented distribution of age, sex, PY and CY of all the samples recruited in the discovery and replication phase in online Table 1 and 2, respectively. We found that some of the parameters differed significantly in different comparison groups. We, therefore, adjusted age, sex and tobacco habit in all the association tests by logistic regression. However, to assess the contribution of tobacco exposure to disease predisposition, we also performed association test without its adjustment after dividing the subjects into high and low dose groups with discovery phase samples.

### DNA Repair Gene Variants Confer Risk/Protection to OSCC and Leukoplakia

In discovery phase, some of the genotyping data were removed due to following reasons: (i) 13 individuals with <92% genotyping calls, (ii) 6 SNPs with <90% genotyping calls, (iii) 18 SNPs removed based on Hardy-Weinberg test with P-Value <0.001, and (iv) 108 SNPs removed for MAF <0.05. In the final analysis more than 98% genotyping rate was observed with 195 SNPs in 336 OSCC, 239 leukoplakia and 512 control samples. The genomic inflation factor (λ) of the QC dataset was 1 to 1.01. We found that the power of the study is 81%, which is considered as sufficient for the identification of associations.

Table 3 provides P-values for different association tests such as: (a) without any adjustment of covariates [age, sex and tobacco habit by logistic regression] and corrections for multiple testing [Benjamini-Hochberg FDR for multiple testing], (b) without any covariate adjustment but with correction for multiple testing, (c) with covariate adjustments but no multiple testing correction and (d) with both covariate adjustments and multiple testing correction. We found rs12515548 of *MSH3* to be significantly associated with the CC group [P-value 7.83E-03, OR: 1.733 (1.333–2.254)]. Significance of this association increased when comparison was made separately between oral cancer and control (Table 3). Another SNP rs207943 of *XRCC5* also showed significant association with oral cancer. Interestingly, these two SNPs were also found to be significantly associated with OSCC when compared to leukoplakia samples as control (Table 3). These results suggest that they have strong influence on predisposition to oral cancer whether or not they are presented as premalignant lesions. Two other loci (rs7003908 of *PRKDC* and rs12360870 of *MRE11A*) showed exclusive associations with leukoplakia; one being risk (rs12360870) and the other protective (rs7003908). The significant allelic association of rs12515548, rs207943 and rs12360870 also remained significant at the genotypic level (Table S1).

**Table 3:** Allelic association results among different comparison groups.

Gene	SNP(Major/Minor Allele)	MAF^a^	Test^b^	OR (95% CI)	P-value			
					Un-adjusted & Un-corrected^c^	Un-adjusted but Corrected^d^	Adjusted but un-corrected^e^	Adjusted & Corrected^f^
MSH3	rs12515548(A/G)	0.096	CC	1.733 (1.333–2.254)	2.87E-05	5.60E-03	4.01E-05	7.83E-03
		0.096	CAC	2.231(1.666–2.988)	6.78E-09	1.32E-06	7.32E-08	1.43E-05
		0.104	CAL	2.234 (1.52–3.282)	2.38E-05	1.61E-03	4.25E-05	2.87E-03
XRCC5	rs207943(C/G)	0.364	CAC	1.734 (1.412–2.129)	4.07E-08	3.96E-06	1.47E-07	1.43E-05
		0.345	CAL	1.84 (1.431–2.366)	3.63E-07	3.69E-05	1.97E-06	2.00E-04
MRE11A	rs12360870(G/A)	0.290	LC	1.9 (1.545–2.337)	3.54E-12	6.91E-10	7.72E-09	2.37E-07
PRKDC	rs7003908(A/C)	0.107	LC	0.218 (0.119–0.399)	4.90E-06	4.77E-04	9.60E-08	7.99E-05

a MAF: Minor Allele Frequency of reference population is listed;

b Association tests abbreviations, CC: case (jointly oral cancer and leukoplakia) vs. control, CAC: cancer vs. control, CAL: cancer vs. leukoplakia and LC: leukoplakia vs. control;

c P-values without any adjustment for age, sex and tobacco habits by logistic regression and without any multiple tests correction applied,

d P-values without any adjustment for age, sex and tobacco habits by logistic regression but corrected for multiple testing by Benjamini-Hochberg False Discovery Rate method,

e P-values after adjustment for age, sex and tobacco habits by logistic regression but no correction multiple testing was applied,

f P-values after adjustment for age, sex and tobacco habits by logistic regression and corrected for multiple testing by Benjamini-Hochberg False Discovery Rate method.

We performed stratification analysis to verify the confounding effect of evolutionary genetic heterogeneity within the studied population on the association results. Similar clustering was observed on both cases and controls (Fig. S1). Interestingly, similar clustering was also observed when analysis was done based on sample type (i.e. OSCC, leukoplakia and controls, Fig. S1) or geographical locations (Fig. S1). We next performed association test in CC group using first four principal components as covariates. The SNP rs12515548 of the *MSH3* remained significant [allelic association P-value: 0.006, OR: 1.1717 (1.318–2.236)] as it was observed without the stratification adjustment. We continued this analysis in all four groups (CC, CAC, LC and CAL) and found that no associated variants were excluded due to the observed clustering (Table S2).

### Tobacco Exposure Modifies the Effect of DNA Repair Gene Variants on Oral Cancer and Leukoplakia Predisposition

We performed association analysis using tobacco exposure as covariate to better understand its role in oral cancer and leukoplakia in the discovery phase samples. Table 4 shows that most of the comparative groups exhibited association with the low-dose (LD) tobacco exposure level. The two significantly associated SNPs with OSCC (rs12515548 and rs207943) also showed significant association with low-dose tobacco exposure group. Interestingly, these two SNPs also showed association with low dose tobacco group when compared between cancer and leukoplakia where leukoplakia was considered as reference (CAL-LD in Table 4). Carriers of two SNPs (rs12360870 of *MRE11A* and rs7003908 of *PRKDC*) continued to show similar effects (one being risk and other protective) on leukoplakia development when exposed to both high and low-dose of tobacco (LC-LD and LC-HD in Table 4). These results suggest their strong role on OSCC predisposition irrespective of tobacco exposure level. Table S3 shows association results at the genotypic level. We found all the significant variants from allelic association remained significant in genotypic tests also, except rs7003908 of *PRKDC*.

**Table 4:** Allelic associations in with respect to tobacco exposure.

Gene	SNP(Major/Minor Alleles)	MAF^a^	Test^b^	OR (95% CI)	P-values^c^
MSH3	rs12515548(A/G)	0.089	CC-HD	1.385 (0.997–1.922)	0.558
			CC-LD	1.837 (1.398–2.413)	2.48E-03
		0.089	CAC-HD	1.718 (1.202–2.456)	0.15
			CAC-LD	3.37(1.893–6.001)	2.48E-06
		0.101	CAL-HD	1.756 (1.097–2.81)	0.568
			CAL-LD	2.251 (1.533–3.303)	3.21E-03
XRCC5	rs207943(C/G)	0.358	CAC-HD	1.505 (1.149–1.972)	0.15
			CAC-LD	1.767 (1.433–2.178)	9.57E-06
		0.336	CAL-HD	1.68 (1.231–2.292)	0.073
			CAL-LD	1.771 (1.38–2.273)	7.30E-04
MRE11A	rs12360870(G/A)	0.279	LC-HD	2.264 (1.702–3.013)	4.09E-06
		0.295	LC-LD	1.796 (1.45–2.224)	1.58E-05
PRKDC	rs7003908(A/C)	0.088	LC-HD	0.162 (0.062–0.427)	0.023
		0.105	LC-LD	0.212 (0.113–0.399)	1.43E-04

a MAF: Minor allele frequency of the reference population is listed;

b Association tests abbreviations, CC: case (jointly oral cancer and leukoplakia) vs. Control, CAC: cancer vs. Control, CAL: cancer vs. Leukoplakia, LC: leukoplakia vs. control, HD: High-dose and LD: Low-dose tobacco exposed group;

c Benjamini-Hochberg False Discovery Rate corrected P-values for multiple tests.

### Validation of Selected SNPs in OSCC-control Replication Cohort

Next, we genotyped rs12515548 of *MSH3* and rs207943 of *XRCC5* in a separate cohort of 114 OSCC patients and 160 control subjects to validate the discovery phase results. The unavailability of a separate cohort of leukoplakia samples prevented us from validation of rs12360870 and rs7003908 that were found to be significantly associated exclusively with leukoplakia samples in the discovery phase. We found only rs12515548 remained significantly associated with OSCC in both allelic and genotypic analysis (replication P-value: allelic 4.83E-03, genotypic 0.044; Table 5 and Table S4). The combined P-values for this SNP of discovery and replication phase for allelic and genotypic tests are 1.21E-06 and 0.009, respectively.

**Table 5:** Allelic association results of replication study and comparison with discovery data.

Gene	SNP(Minor/Major Allele)	Phase of study	MAF^b^	OR (95% CI)	P-value
MSH3	rs12515548(A/G)	Replication	0.097	4.424 (1.572–12.45)	4.84E-03#
		Discovery	0.096	2.231 (1.666–2.988)	1.43E-05*
XRCC5	rs207943(C/G)	Replication	0.363	1.065 (0.474–2.392)	0.8796#
		Discovery	0.364	1.734 (1.412–2.129)	1.43E-05*

a MAF: Minor allele frequency of the reference population is listed;

* Benjamini-Hochberg False Discovery Rate corrected P-values for multiple tests;

# Unadjusted P-values.

### SNP-SNP and SNP-environment Interaction Reveals Moderate Synergistic Effects

We performed MDR analysis to reveal the SNP-SNP and SNP-environment factors interactions in this cohort of individuals. We found the most potent interaction in OSCC as compared with control is between rs207943, rs12515548, Age and tobacco smoking with a TBA of 0.6011 and CVC 10 (p-value 0.001). However, the most significant model for OSCC development form leukoplakia was the interaction among rs207943, rs12515548, sex and tobacco chewing (Table S5). For leukoplakia development from control, the most significant model was the interaction of all covariates with rs12360970 followed by inclusion of rs7003908 (Table S5).

**Figure 2:**
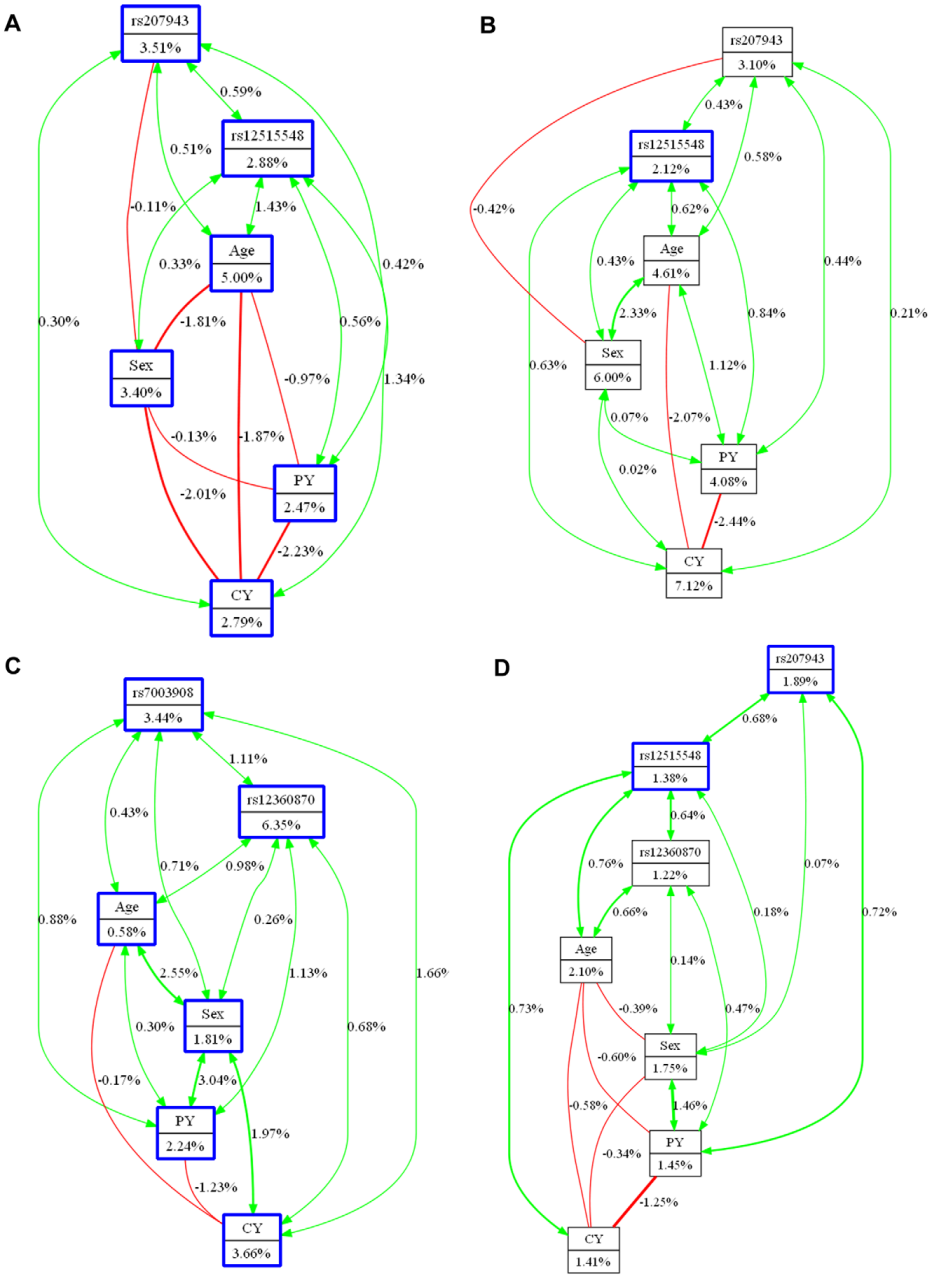
Orange canvas interaction models. These models describe the percent of entropy explanation by single factor or two way interactions. The boxes describe the SNPs and factors with the percentage of entropy explained. Interaction is presented by arrows and redundancy by lines. Interaction models are constructed on (A) oral cancer versus control (CAC), (B) oral cancer versus leukoplakia (CAL), (C) leukoplakia versus control (LC) and (D) case versus control (CC).

Next, we applied interaction entropy algorithms to support interpretation of the relationship between the variables. We found the most potent model of OSCC (CAC) as revealed from permutation testing (rs207943-rs12515548-Age-PY) is synergistic in nature (Fig. 2A). Interestingly, the age and sex contributes to this interaction in an independent manner with an entropy removal of 1.43% and 0.56%, respectively. The synergistic interaction was also observed in the model consisting of rs207943, rs12515548, Sex and CY for OSCC development from leukoplakia (CAL), where all factors work jointly (Fig. 2B). We found age in the CAC comparison and tobaccos chewing in CAL comparison are the most important covariates with 5% and 7.12% entropy removal, respectively. For leukoplakia development rs12360870 is the strongest factor (entropy explained: 6.35%) and all significant interactions are synergistic (Fig. 2C). The model for CC comparison resembled both CAC and LC comparisons (Fig. 2D).

## Discussion

The regional genetic and lifestyle heterogeneity among populations from different parts of India have been noted by many investigators [46]–[48]. This poses serious impediment to the genetic association study in Indian populations. We thus, targeted the middle and low-income group of semi-urban population with an age range of 22 to 80 years from the state of West Bengal in this study. We also ensured similar tobacco habits of the case and control individuals who participated in the study. The ongoing Million Death Study (MDS) in India finds an increase in age-specific cancer risk due to tobacco habit in the population from West Bengal [2]. Another study also reported association of oral habit and DNA damage with OSCC and leukoplakia in these populations [49].

The most promising associated SNP from this study is rs12515548 of *MSH3*. This SNP was found to be significantly associated in three out of four analysis sets tested in the discovery phase (case-control, cancer-control and cancer-leukoplakia) and also remained significant in the replication phase. No association was found with this SNP in GWAS of upper aerodigestive tract cancers and this is the first report of association of this SNP with OSCC. However, several studies showed association of other SNPs in MSH3 and MSH6 genes in different cancers [33], [50], [51]. It may be noted that, although we have observed relatively strong P-values in the association tests for the given sample size, the power of the study is 0.81 and there was no population stratification. However, further replication is essential in same and other populations. The rs12515548 is an intronic SNP located near 21th exon of the *MSH3* with a change from G to A (http://www.ncbi.nlm.nih.gov/projects/SNP/snp_ref.cgi?rs=12515548). Two functional attributes may be associated with this SNP, (a) functionality prediction using F-SNP [52] revealed that it loses the capacity to bind GATA family of transcription factors upon change from G to A (confidence score of binding prediction for different GATA transcription factors ranges from 88.4 to 98.4) and (b) the miRBase analysis showed an increased affinity of hsa-miR-374a-3p to the risk allele (A) of the SNP (score 6.9, evalue 1.0 for allele A; score 60, evalue 5.6 for allele G). Direct experimental validations are needed to understand its exact functional role, if any. The results from Indian Genome Variation Consortium [47] and admixture mapping of Indian population identified the caste populations of the eastern India as Indo-European population which show relatedness to the CEU population of the HapMap [53], [54]. We thus, build a LD map of *MSH3* using imputed data from HapMap CEU population and found an 81 Kb LD block with rs12515548 which includes exon 21 (data not shown). It would be interesting to examine whether or not such LD block exist in this populations and, if so, whether rs12515548 is linked with any other functional SNP of the *MSH3*. The intronic SNP rs207943 of *XRCC5*, which also showed significant association with OSCC development, is present within a putative binding site of the transcription factor Skn-1 of *C. elegans*. It binds only with non-risk G allele of the SNP (F-SNP prediction score 0.5, binding score 87.1). The human homolog of Skn-1, Nrf 1/2/3 is an important transcription factor involved in oxidative stress resistance [55]. The Nrf2 deficient mice have attenuated expressions of many detoxifying and antioxidant enzymes and are highly susceptible to carcinogen induced toxicity and carcinogenesis [56]. Thus, the inability of Skn-1 binding with the risk allele C of this SNP and OSCC progression needs to be investigated further.

The study also probed genetic risk factors associated with the development of leukoplakia and its conversion to OSCC. We found different SNPs to be associated exclusively with the development of leukoplakia from normal individuals and progression of leukoplakia to cancer. For example, rs7003908 of *PRKDC* was reported to be associated with prostate and urinary bladder cancer in north-Indian populations and glioblastoma in United States [57]–[59]. Identification of a specific risk SNP associated with cancer-leukoplakia comparison would be valuable as a prognostic biomarker for the detection of cases where leukoplakia would have the potential of conversion to oral cancer. However, replication of the association in another cohort of leukoplakia patients is required to validate these results.

The tobacco exposure is a known environmental factor associated with oral cancer and leukoplakia. Thus, we performed association test without its adjustment and stratifying the subjects based on their tobacco exposure levels. The observation that a few polymorphic variants of DNA repair and damage response genes exhibited association to a different tobacco exposed groups suggests that DNA damage signals are differentially processed by different polymorphic variants of these genes. Similar observation has also been made in previous studies with *p53* gene polymorphisms [28]. It may be noted that these SNPs might be useful for development of tobacco-associated predictive marker for oral cancer and leukoplakia. The MDR analysis revealed age in OSCC and chewing in leukoplakia are the two important covariates which interacts synergistically with the most potent risk SNPs of the respective diseases (rs12515548 and rs207943 for OSCC and rs12360870 for leukoplakia). The study revealed synergy between SNPs and redundancy between lifestyle factors albeit without any additive effect. This particular phenomena was also observed with the SNPs from DNA repair genes in other caner types [60]. Thus, it may be suggested that the overall repair capacity contributed by different repair machineries and independent effects of various lifestyle factors are the ultimate determinant of oral cancer and leukoplakia predisposition in an individual.

The present study suggests that *MSH3, XRCC5, MRE11A* and *PRKDC* to be the four most important genes that would modify the risk of predisposition to oral cancer and leukoplakia in these eastern Indian populations. Polymorphic variants of these genes were found to be significantly associated with breast, pancreatic, colorectal and ovarian cancers [61]–[64]. However, to the best of our knowledge, none of the variants identified in this study were previously reported to be associated with any other cancer, except rs7003908. MSH3 upon phosphorylation by ATM/ATR initiates DNA mismatch repair with MSH2 and directs downstream MMR events, including strand discrimination, excision, and re-synthesis with MLH1 and PMS1 [36], [65]. XRCC5 with XRCC6 forms a dimer and increases the affinity of PRKDC, the catalytic subunit of *DNA-PK* [DNA-dependent serine/threonine protein kinase] [66]. It plays several crucial roles like, recognition and recruitment of other components to DSB and phosphorylation of several transcription factors including p53 [67]. Several other phosphorylating substrates of PRKDC have also crucial role in cancer, like, c-Myc, PARP, c-JUN [68]–[70]. MRE11A, one of the partners of MRE11A-RAD50-NBN complex involved in DSB repair, have also role in telomerase integrity and meiosis. The functional implications of either the associated intronic SNPs or their linked functional SNPs in these genes are needed to be investigated in future.

## Supporting Information

Figure S1 **Population stratification analysis.** Similar clustering was observed in principal component analysis (A) in case and controls, (B) in leukoplakia, controls and cancer and (C) in different geographical locations.
(TIF)

Table S1 **Genotypic association results among different comparison groups.**
(DOC)

Table S2 **Estimated P Values of allelic association tests after adjustment of first four principal components.**
(DOC)

Table S3 **Genotypic association results among different comparison groups with respect to tobacco exposure.**
(DOC)

Table S4 **Genotypic results of replication study and comparison with discovery data.**
(DOC)

Table S5 **MDR interaction analysis between SNPs and lifestyle factors.**
(DOC)

Methods S1 **Supplementary methods.**
(DOC)
