# Supplementary figures and images for: Comprehensive SNP Scan of DNA Repair and DNA Damage Response Genes Reveal Multiple Susceptibility Loci Conferring Risk to Tobacco Associated Leukoplakia and Oral Cancer
Source: PLoS One. 2013 Feb 20;8(2):e56952. doi: 10.1371/journal.pone.0056952 (PMC3577702; doi:10.1371/journal.pone.0056952)

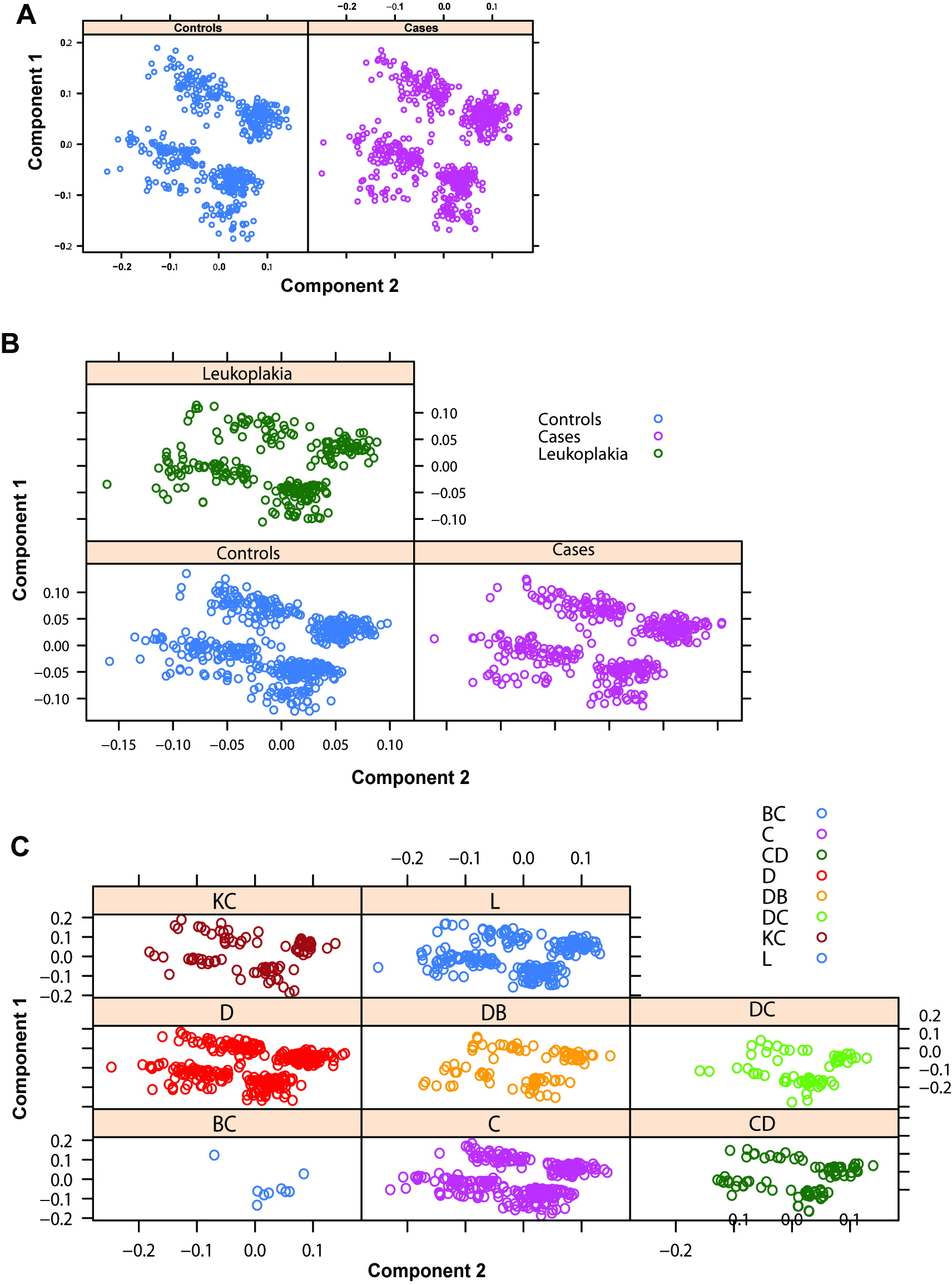

Supplement: Figure S1 — Population stratification analysis. Similar clustering was observed in principal component analysis (A) in case and controls, (B) in leukoplakia, controls and cancer and (C) in different geographical locations. (TIF) [file pone.0056952.s001.tif]
