# Supplementary material for: Comprehensive SNP Scan of DNA Repair and DNA Damage Response Genes Reveal Multiple Susceptibility Loci Conferring Risk to Tobacco Associated Leukoplakia and Oral Cancer
Source: PLoS One. 2013 Feb 20;8(2):e56952. doi: 10.1371/journal.pone.0056952 (PMC3577702; doi:10.1371/journal.pone.0056952)
Supplement: Table S1 — Genotypic association results among different comparison groups. (DOC) [file pone.0056952.s002.doc]

**Supplementary Table S1. Genotypic association results among different comparison groups**

| **Gene** | **SNP   (Minor/ Major Alleles)** | **Test ^a^** | **Genotypes^b^** | **Genotype Counts** | | **OR (95% CI)** | **P ^c^** |
| --- | --- | --- | --- | --- | --- | --- | --- |
|  |  |  |  | **Affected** | **Unaffected** |  |  |
| MSH3 | rs12515548  (A/G) | CAC | AA/AG/GG | 17/98/222 | 5/90/419 | 2.631 (1.563-4.429) | 0.026 |
| XRCC5 | rs207943 (C/G) | CC | CC/CG/GG | 136/240/217 | 60/251/203 | 1.434 (1.194-1.72) | 0.021 |
|  |  | CAC |  | 98/143/102 | 60/251/203 | 1.842 (1.495-2.269) | 1.82E-06 |
|  |  | CAL |  | 98/143/102 | 38/97/115 | 1.851 (1.432-2.391) | 5.15E-04 |
| MRE11A | rs12360870  (G/A) | CC | GG/GA/AA | 115/190/281 | 58/181/275 | 1.403 (1.17-1.683) | 0.025 |
|  |  | LC |  | 82/65/96 | 58/181/275 | 2.03 (1.636-2.519) | 2.48E-08 |

^a^ Association tests abbreviations, CC: case (jointly oral cancer and leukoplakia) vs. control, CAC: cancer vs. control, CAL: cancer vs. leukoplakia and LC: leukoplakia vs. control; ^b^ genotypes (minor homozygote/heterozygote/reference homozygote); ^c^ Benjamini-Hochberg False Discovery Rate corrected P values for multiple tests.
