# Supplementary material for: Comprehensive SNP Scan of DNA Repair and DNA Damage Response Genes Reveal Multiple Susceptibility Loci Conferring Risk to Tobacco Associated Leukoplakia and Oral Cancer
Source: PLoS One. 2013 Feb 20;8(2):e56952. doi: 10.1371/journal.pone.0056952 (PMC3577702; doi:10.1371/journal.pone.0056952)
Supplement: Table S2 — Estimated P Values of allelic association tests after adjustment of first four principal components. (DOC) [file pone.0056952.s003.doc]

**Supplementary Table S2.** Estimated P Values of allelic association tests after adjustment of first four principal components

| **Gene** | **SNP** | **P-Value** | **Test** |
| --- | --- | --- | --- |
| MSH3 | rs12515548 (A/G) | 0.006154 | CC |
|  |  | 8.00E-06 | CAC |
|  |  | 0.001583 | CAL |
| XRCC5 | rs207943 (C/G) | 1.10E-05 | CAC |
|  |  | 0.0008093 | CAL |
| MRE11A | rs12360870 (G/A) | 3.67E-07 | LC |
| PRKDC | rs7003908 (A/C) | 6.58E-05 | LC |
