# Supplementary material for: Comprehensive SNP Scan of DNA Repair and DNA Damage Response Genes Reveal Multiple Susceptibility Loci Conferring Risk to Tobacco Associated Leukoplakia and Oral Cancer
Source: PLoS One. 2013 Feb 20;8(2):e56952. doi: 10.1371/journal.pone.0056952 (PMC3577702; doi:10.1371/journal.pone.0056952)
Supplement: Table S3 — Genotypic association results among different comparison groups with respect to tobacco exposure. (DOC) [file pone.0056952.s004.doc]

**Supplementary Table S3.** Genotypic association results among different comparison groups with respect to tobacco exposure

| **Gene** | **SNP  (Minor/Major Alleles)** | **Genotypes ^a^** | **Test ^b^** | **Genotype Counts** | | **OR (95% CI)** | **P ^c^** |
| --- | --- | --- | --- | --- | --- | --- | --- |
|  |  |  |  | **Affected** | **Unaffected** |  |  |
| MSH3 | rs12515548  (A/G) | AA/AG/GG | CAC-HD | 13/49/131 | 3/60/229 | 2.685 (1.408-5.118) | 0.135 |
|  |  |  | CAC-LD | 17/95/207 | 5/78/401 | 2.627 (1.56-4.423) | 0.027 |
| XRCC5 | rs207943 (C/G) | CC/CG/GG | CC-HD | 81/132/126 | 34/159/99 | 1.336 (1.049-1.702) | 0.113 |
|  |  |  | CC-LD | 124/227/203 | 55/234/195 | 1.449 (1.2-1.75) | 0.024 |
|  |  |  | CAC-HD | 55/81/59 | 34/159/99 | 1.63 (1.24-2.144) | 0.094 |
|  |  |  | CAC-LD | 94/136/95 | 55/234/195 | 1.89 (1.526-2.341) | 1.08E-06 |
|  |  |  | CAL-HD | 55/81/59 | 23/48/67 | 1.653 (1.207-2.264) | 0.354 |
|  |  |  | CAL-LD | 94/136/95 | 32/90/107 | 1.803 (1.393-2.333) | 1.49E-03 |
| MRE11A | rs12360870 (G/A) | GG/GA/AA | LC-HD | 48/41/44 | 31/100/160 | 2.418 (1.808-3.234) | 5.19E-07 |
|  |  |  | LC-LD | 75/60/88 | 57/172/255 | 1.955 (1.574-2.427) | 2.53E-07 |

^a^ Genotypes (minor homozygote/heterozygote/reference homozygote); ^b^ Association tests abbreviations, CC: case (jointly oral cancer and leukoplakia) vs. control, CAC: cancer vs. control, CAL: cancer vs. leukoplakia and LC: leukoplakia vs. control, HD: high dose and LD: low dose of tobacco exposure; ^c^ Benjamini-Hochberg False Discovery Rate corrected P values for multiple tests.
