# Supplementary material for: Comprehensive SNP Scan of DNA Repair and DNA Damage Response Genes Reveal Multiple Susceptibility Loci Conferring Risk to Tobacco Associated Leukoplakia and Oral Cancer
Source: PLoS One. 2013 Feb 20;8(2):e56952. doi: 10.1371/journal.pone.0056952 (PMC3577702; doi:10.1371/journal.pone.0056952)
Supplement: Table S4 — Genotypic results of replication study and comparison with discovery data. (DOC) [file pone.0056952.s005.doc]

**Supplementary Table S4.** Genotypic results of replication study and comparison with discovery data

| **SNP (Minor/Major Alleles)** | **Phase of study** | **Genotypic counts ^a^** | | **OR(95% CI)** | **P-value** |
| --- | --- | --- | --- | --- | --- |
|  |  | **Affected** | **Unaffected** |  |  |
| rs12515548 (A/G) | Discovery | 17/98/222 | 5/90/419 | 2.631 (1.563-4.429) | 0.026^*^ |
|  | Replication | 3/34/75 | 1/27/121 | 3.878(1.033-14.56) | 0.044^#^ |
| rs207943 (C/G) | Discovery | 98/143/102 | 60/251/203 | 1.842 (1.495-2.269) | 1.82E-06^*^ |
|  | Replication | 19/73/20 | 25/66/69 | 1.109(0.6046-2.032) | 0.739^#^ |

^a^ The genotypic counts are for minor allele homozygote/heterozygote/major allele homozygote; ^*^ Benjamini-Hochberg False Discovery Rate corrected P values for multiple tests; ^#^ Unadjusted P-values
