# Supplementary material for: Comprehensive SNP Scan of DNA Repair and DNA Damage Response Genes Reveal Multiple Susceptibility Loci Conferring Risk to Tobacco Associated Leukoplakia and Oral Cancer
Source: PLoS One. 2013 Feb 20;8(2):e56952. doi: 10.1371/journal.pone.0056952 (PMC3577702; doi:10.1371/journal.pone.0056952)
Supplement: Table S5 — MDR interaction analysis between SNPs and lifestyle factors. (DOC) [file pone.0056952.s006.doc]

**Supplementary Table S5.** MDR interaction analysis between SNPs and lifestyle factors

| **MDR models** | **TBA ^a^** | **CVC ^b^** | **P Value ^c^** | **Group** |
| --- | --- | --- | --- | --- |
| rs207943-rs12515548-rs12360870-Age-Sex-PY | 0.5816 | 10 | 0.001 | CC |
| rs207943-rs12515548-Age-PY | 0.6011 | 10 | 0.001 | CAC |
| rs207943-rs12515548-Sex-CY | 0.6118 | 10 | 0.001 | CAL |
| rs12360870-Age-Sex-PY-CY | 0.6051 | 10 | 0.001 | LC |
| rs12515548-rs12360870-Age-Sex-PY-CY | 0.6013 | 10 | 0.002 | LC |

^a^ TBA: Testing Balance Accuracy; ^b^ CVC: Cross Validation Consistency; ^c^ P-values as calculated after 1000 permutations.
