# Supplementary material for: Comprehensive SNP Scan of DNA Repair and DNA Damage Response Genes Reveal Multiple Susceptibility Loci Conferring Risk to Tobacco Associated Leukoplakia and Oral Cancer
Source: PLoS One. 2013 Feb 20;8(2):e56952. doi: 10.1371/journal.pone.0056952 (PMC3577702; doi:10.1371/journal.pone.0056952)
Supplement: Methods S1 — Supplementary methods. (DOC) [file pone.0056952.s007.doc]

**Supplementary Methods S1**

We have used the algorithm developed by Sarkar-Roy et al. (2011). The methodology is reproduced below with permission of the authors. The algorithm selects a subset of “maximally informative” SNPs from among the SNPs cataloged in the HapMap database, with following three characteristics: (a) high heterozygosity, with minor allele frequencies as close to 0.5 as possible in the pool of data from the 4 HapMap populations, (b) low linkage-disequilibrium values with adjacent SNPs, and (c) the distance between selected SNPs does not exceed a user-defined, pre-specified value.

The algorithm comprises the following steps:

*Step 1*: Identify the start and end nucleotide positions of the genomic region from which SNPs are to be selected.

*Step 2*: From HapMap (www.hapmap.org) and HapMart download data on all polymorphic SNPs (i.e., SNPs with MAFs >0.05) in each of the 4 HapMap populations pertaining to the chosen genomic region. For each population, the downloaded data include (a) Reference allele frequency, (b) Heterozygosity, and (c) LD value with the preceding SNP.

*Step 3*: Chose the SNP from the HapMap data of the 4 populations that is closest to the starting nucleotide position of the chosen genomic region, called the anchor SNP, as the first SNP to be included in the subset.

*Step 4*: Define two distances (in kb): (a) window-width (W), and (b) jump-distance (D). The motivation for defining these distances is to select “informative” SNPs from windows within the genomic region of a defined width (W) separated by a defined distance (D). This is because it is expected *a priori* that SNPs in close proximity will have high LD values in any of the HapMap populations. Of course, full coverage of the genomic region is ensured by setting D=0.

*Step 5*: For every SNP within a window cataloged in the HapMap database, extract (a) Reference allele frequency, (b) Heterozygosity, and (c) LD value with the preceding SNP, from the data downloaded from HapMap and HapMart.

*Step 6*: Calculate the averages over HapMap populations of these three values for the window under consideration. We note that while the number of HapMap populations, n_1_ [=1 (CEU), 2 (CHB), 3 (JPT), 4 (YRI)], for which data on reference allele frequencies and heterozygosities for a SNP are available is the same, the sample size n_2_ (1< n_2_ <4) for estimation of LD between a pair of SNPs may be different from n_1_.

*Step 7*: Select a SNP from among the SNPs identified in a window. In order to select a SNP, for each SNP in the window, compute a Score (S) defined as:

$S=\frac{\sum_{i=1}^{n_{1}} \left|p_{i}-\left.0.5\right|\right.}{n_{1}}+\frac{r^{2}}{n_{2}}$,

where *p_i_* = reference allele frequency in the *i*-thHapMap population,

*n*_1_ = number of HapMap populations for which allele frequency data were available,

*r*^2^ = average LD with previous selected SNP based on *n*_2_ HapMap populations.

The motivations underlying the definition of S are that we wish to select the SNP that is most polymorphic, i.e., reference allele frequency closest to 0.5, and in least LD with the previously selected SNP. A SNP satisfying both characteristics will have a small value of S. However, no SNP in the window may satisfy both these requirements. Therefore, in defining S, we have chosen to take a linear combination of the averages of the differences of reference allele frequencies in the HapMap populations from 0.5 and of the LD with the previous SNP selected. However, (a) since the values of n_1_ and n_2_ may be different, a linear combination of *weighted* averages, weighted by the corresponding sample sizes, needs to be taken; and, (b) since averages based on a higher number of populations will be more stable, higher weightage needs to be given to an average based on a larger number of populations. From among the SNPs in the window, the SNP with the *minimum* value of S is selected.

Sarkar-Roy N, Mondal D, Bhattacharya P, Majumder PP (2011) A Novel Statistical Algorithm for Maximizing the Utility of HapMap Data to Design Genomic Association Studies in non-HapMap Populations. *Int. J. Data Mining and Bioinformatics.* 5: 706-716.
